# *PAX6* analysis of one family and one sporadic patient from southern China with classic aniridia

**Published:** 2011-11-26

**Authors:** Ying Lin, Xialin Liu, Xuanwei Liang, Baoxin Li, Shuhong Jiang, Shaobi Ye, Huiqin Yang, Bingsheng Lou, Yizhi Liu

**Affiliations:** 1 State Key Laboratory of Ophthalmology, Zhongshan Ophthalmic Center, Sun Yat-sen University, Guangzhou, China; 2 Department of Pharmacology (State-Province Key Laboratories of Biomedicine-Pharmaceutics of China), Harbin Medical University, Harbin, Heilongjiang, China; 3 People’s Hospital of Inner Mongolia, Hohhot, China

## Abstract

**Purpose:**

To investigate the paired box gene 6 (*PAX6*) in three patients from southern China presenting with classic aniridia: two patients from two successive generations of one family and one sporadic patient.

**Methods:**

All the available members from two successive generations of one family and one sporadic patient underwent complete physical and ophthalmic examinations. Genomic DNA was extracted from leukocytes of peripheral blood collected from the two generations of family members, the sporadic patient and 100 unrelated control subjects from the same population. Exons 1–13 of the *PAX6* gene were amplified by polymerase chain reaction (PCR) and sequenced directly. The ophthalmic examinations included best-corrected visual acuity, slit-lamp examination, fundus examination, optical coherence tomography, and Pentacam and Goldmann perimetry.

**Results:**

The three patients were affected with aniridia accompanied by microcornea, microphthalmia, and nystagmus. A heterozygous *PAX6* frameshift mutation, c.891del A(p.Gln297HisfsX68) in exon 10, was identified in the affected individuals and not in any of the unaffected family members, including the unaffected family members of the proband patient’s generation. One novel mutation, c.607C>T(Arg203X) in exon 8, was detected in the unrelated sporadic patient.

**Conclusions:**

Although *PAX6* gene mutations and polymorphisms have been reported from various ethnic groups, we report for the first time the identification of two new *PAX6* gene mutations in Chinese aniridia patients.

## Introduction

Congenital aniridia is a rare ocular disease that affects the development of multiple ocular structures; this abnormality is usually caused by mutations in the paired box gene 6 (*PAX6*) located on chromosome 11p13 [1-5]. The disease is characterized by a lack of iris and accompanied by other symptoms, including severe age-related corneal degeneration, cataract, and nystagmus [6-10].

At present, the PAX6 mutation database lists more than 300 different mutations of that gene [3,11-17]. This study analyzes the coding sequences of *PAX6* in two patients with aniridia from successive generations of one Chinese family and one sporadic patient with aniridia. Two novel mutations in *PAX6* were detected in the Chinese population; both mutations were heterozygous.

## Methods

### The aniridia family

**Figure 1:**
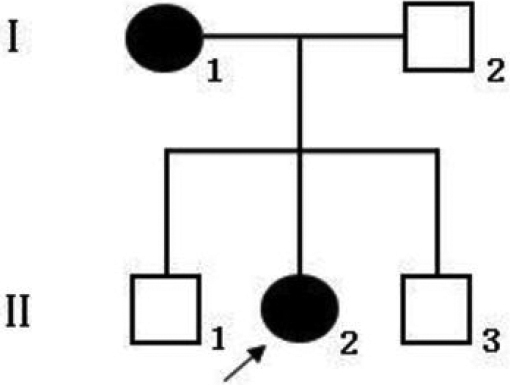
The pedigree of Chinese Family 1 with aniridia. Square symbols denote males and circle symbols denote females. The shaded symbols indicate individuals with ophthalmologist-confirmed aniridia. The arrow points to the proband.

One family (Figure 1) and one sporadic patient were diagnosed at the Zhongshan Ophthalmic Center as having aniridia.

We performed the ophthalmic examinations as follows:

● Visual acuity was examined using the ETDRS chart (Precision Vision, La Salle, IL).
● Anterior segment photograph was obtained using a BX 900 Slit Lamp (Haag-Streit, Bern, Switzerland).
● A fundus photograph was obtained using a Heidelberg Retina Angiograph (Heidelberg Engineering, Heidelberg, Germany).
● Optical coherence tomography (OCT) scans (TOPCON, Tokyo, Japan) were used to assess the thickness and pathology of the posterior pole of the retina.
● Anterior segment dimensions were measured with Pentacam HR version 70700 (Oculus, Weltzar, Germany).

In addition, we performed complete physical examinations of the patients to exclude systemic diseases.

### Sample collection

The two generations of one affected family and one sporadic patient were identified at the Zhongshan Ophthalmic Center. One hundred subjects (25.34±8.63 years old, 45 male) from the same population without diagnostic features of aniridia were recruited to serve as normal controls. After obtaining informed consent from all participating individuals following the principles of the Declaration of Helsinki, peripheral venous blood samples were collected for genomic DNA extraction from the blood leucocytes using standard protocols.

### Detection of the mutation

All coding exons of the known candidate gene (*PAX6*) and their flanking regions were amplified by polymerase chain reaction (PCR) using the primers in Table 1 [18]. Briefly, PCR was conducted in 50 μl reactions and cycling profile included one cycle of 94 °C for 5 min followed by 40 cycles of 94 °C for 45 s, 52-66 °C (please see Table 1 for annealing temperature and other details) for 45 s, and 72 °C for 45 s, and one cycle of 72 °C for 10 min. The PCR products were sequenced from both directions with the ABI3730 Automated Sequencer (PE Biosystems, Foster City, CA). The sequencing results were analyzed using Chromas (version 2.3; Technelysium Pty Ltd, Brisbane, Australia) and compared with the reference sequences in the database at the National Center for Biotechnology Information (NCBI; NC_000011.9). The superimposed mutant PCR products were sub-cloned into pGEM-T vector (Promega, Madison, WI) and sequenced to identify the mutation. Briefly, PCR products were purified by gel extraction using gel extraction kits (AXYGEN, China) according to the manufacturer's instructions. The purified PCR fragments were ligated into the pGEM-T easy vector (Invitrogen) and the resulting plasmids were transfected by heat shock into DH5a competent *Escherichia coli* for propagation. Glycerol stocks were frozen to maintain the clones. Colonies were picked and grown overnight in 1-2 ml of Luria-Bertani broth containing ampicillin (50 mg/ml). Plasmids were purified using the DNeasy Miniprep kit (Qiagen, China). The plasmid DNA was sequenced using the ABI3700 and the T7 primer. Sequences were determined using the DNAman software analysis system.

**Table 1:** Primers used for PCR.

**Exon**	**Forward (5′-3′)**	**Reverse (5′-3′)**	**Product size (bp)**	**Annealing temperature (°C)**
4	TGCAGCTGCCCGAGGATTA	GCACCCCGAGCCCGAAGTC	144	66
5	TCCCTCTTCTTCCTCTTCACT	GGGGTCCATAATTAGCATC	301	58
5 a,6	GCTCTCTACAGTAAGTTCTC	AGGAGAGAGCATTGGGCTTA	457	59
7	AATCCACCCACTGTCCCG	CCAGCCACCTTCATACCG	542	60
8	TCAGGTAACTAACATCGCA	GTTGACTGTACTTGGAAGAA	719	53
9,10,11	GAGGTGGGAACCAGTTTGATG	CAAGCCAATCTCTGTAGTGCG	890	52
12	GCTGTGTGATGTGTTCCTCA	AAGAGAGATCGCCTCTGTG	245	58
13	CATGTCTGTTTCTCAAAGGG	CCATAGTCACTGACTGAATTAACAC	202	58

Summary of the primers and product length used for the amplification of *PAX6* exons.

## Results

### Clinical data

**Figure 2:**
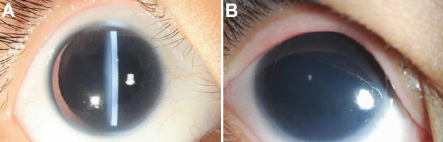
Ocular photographs of patient II-2 (**A**) and the sporadic patient 3 (**B**) with aniridia. The two patients each had aniridia and bilateral corneal degeneration, neovasculation, eyeball horizontal tremor, and microphthalmia.

The two generations of the Chinese family and another sporadic patient (case 3) studied in this report originated from the southern area of China. Two individuals in two successive generations (case I.1 and case II.2) and case 3 were found to have the same congenital ocular disease (Figure 2).

The three patients had aniridia and bilateral corneal degeneration, neovasculation, eyeball horizontal tremor, and microphthalmia. They showed abnormal vision even from their early childhood. When this study was performed, the patients could detect only hand movement.

**Figure 3:**
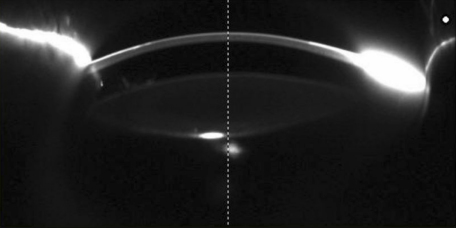
Pentacam photo showing the anterior segment picture of patientII-2.

Applanation tonometry revealed normal intraocular pressures in both eyes. Corneas were unnormal in size and transparency. The width of the cornea of patient II-2 (8 years old, female) was 9.5 mm (OD) and 10.0 mm (OS).The refractive error of patient II-2 was −1.25 diopters (D; OD) and −2.25 D (OS). The axial length of the eyes of patient II-2 was 22.82 mm (OD) and 22.84 mm (OS). No abnormalities were detected in the lens and retina, choroids, and optic nerve. The anterior segment photograph is shown in Figure 3; the anterior chamber depth was 1.77 mm (OD) and 1.84 mm (OS).

The width of the cornea for the case 3 patient (5 years old, male) was 8.5 mm (OD) and 9 mm (OS).The refractive error of the case 3 was +4.50 D (OD) and +6.00 D (OS). The axial length of the eyeballs in case 3 was 19.90 mm (OD) and 19.70 mm (OS). No abnormalities were detected in the lens and retina, choroids, and optic nerve. The anterior chamber depth was 1.89 mm (OD) and 1.99 mm (OS).

### Mutation screening

**Figure 4:**
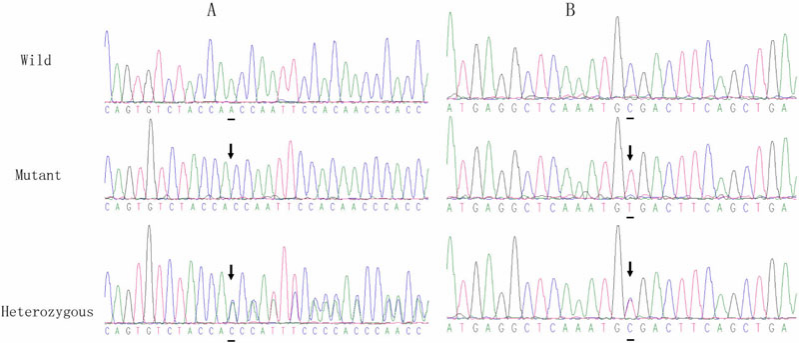
DNA sequence of a part of *PAX6* in the affected patients and unaffected individuals. **A**: A heterozygous G deletion at nucleotide 891 (c.891delA) in exon 10 of *PAX6*. A reading frame shift was observed by sequencing the PCR products of *PAX6* exon 10 in the aniridia patients (Heterozygous). The sequence of the normal allele of exon 10, subcloned into the pGEM-T vector, was used as a control (Wild). A deletion of one nucleotide (A) at nucleotide 891 in exon 10 was identified in the same way (Mutant). **B**: One novel mutation c.607C>T(Arg203X) in exon 8.

In the two affected cases within the families, a heterozygous A deletion at nucleotide 891 (c.891delA) in exon 10 of *PAX6* was confirmed by sequencing (Figure 4A). This frameshift mutation is predicted to cause a premature termination codon (PTC) 68 codons downstream from the ﬁrst new inappropriate codon 297 created by the nucleotide deletion (p.Gln297HisfsX68).

One novel mutation c.607C>T(Arg203X) in exon 8 was detected in the sporadic patient (Figure 4B). No mutations were found in any of the unaffected family members, including the unaffected members of the patient’s generation.

## Discussion

In this study, we found two mutations, one in each of the two exons of *PAX6* that are associated with aniridia: c.891delA and c.607C>T. These two mutations, rather than a rare polymorphism in the normal population, are the causative mutations in the family and the sporadic patient, respectively.

The novel characteristic of the c.891delA mutation identiﬁed in *PAX6* of the family members in the present study is that it occurred at a non-hotspot for mutations. The location is consistent with this mutation’s presence for the ﬁrst time in *PAX6* of Chinese patients.

R203X, like R240X, was previously reported in more than 10 unrelated aniridia patients [19], but this is the first mutation reported in two generations of related Chinese patients. Both mutations are among the most common base changes found in *PAX6*.

The affected members from the family and the sporadic patient had almost the same phenotypes, but different novel mutations. Different phenotypes caused by the same *PAX6* mutation have also been reported previously [20]. These anomalies complicate analyzing the correlation between genotypes and phenotypes [21,22].

In summary, this study identified two novel mutations of *PAX6* in a Chinese family and a sporadic patient with aniridia. This finding expands the mutation spectrum of *PAX6* and is useful and valuable for genetic counseling and prenatal diagnosis in families where aniridia appears, accompanied by corneal degeneration, neovasculation, eyeball horizontal tremor, and microphthalmia.
